# Word Formation Is Aware of Morpheme Family Size

**DOI:** 10.1371/journal.pone.0093978

**Published:** 2014-04-04

**Authors:** Daniela Barbara Keller, Jörg Schultz

**Affiliations:** Department of Bioinformatics, Biocenter, University of Würzburg, Würzburg, Germany; Max Planck Institute for the Physics of Complex Systems, Germany

## Abstract

Words are built from smaller meaning bearing parts, called morphemes. As one word can contain multiple morphemes, one morpheme can be present in different words. The number of distinct words a morpheme can be found in is its family size. Here we used Birth-Death-Innovation Models (BDIMs) to analyze the distribution of morpheme family sizes in English and German vocabulary over the last 200 years. Rather than just fitting to a probability distribution, these mechanistic models allow for the direct interpretation of identified parameters. Despite the complexity of language change, we indeed found that a specific variant of this pure stochastic model, the second order linear balanced BDIM, significantly fitted the observed distributions. In this model, birth and death rates are increased for smaller morpheme families. This finding indicates an influence of morpheme family sizes on vocabulary changes. This could be an effect of word formation, perception or both. On a more general level, we give an example on how mechanistic models can enable the identification of statistical trends in language change usually hidden by cultural influences.

## Introduction

Languages change. This change happens on levels as different as phonology, grammar and the vocabulary, to name just a few. For the speakers of a language, vocabulary change might be one of the most visible processes, as it happens on a comparably small time scale [1]. As words are lost from a language, new ones can emerge. New words can be based on the new association of a string to a meaning, they can be loaned from another language [2] or they can be derived from already existing words. Arguably, the latter is the most frequent process in current Indo-European languages [3]. It can be broken down into two types, namely derivation which changes the syntactic class of a word (e.g. animal → animalish) and compounding, which joins two words (earthquake). Fundamental for the understanding of these two processes is the concept of morphemes, minimal structural and meaning bearing parts of words. The description of how morphemes can be combined to build words has a long standing tradition and comprises a field of linguistics on its own, morphology [4]. But, there is more to morphology than just structure of words. From a completely different viewpoint morphology is also important in the production and perception of words. Classical psycholinguistic experiments revealed that in the process of recognition complex words are decomposed morphologically [5], [6]. Accordingly it was proposed that morphemes are represented in the mental lexicon, the human word store [7]. To understand, how language change influences morphemes, we have recently traced their history in German and English over 200 years. As one result, we found that new words are preferentially built with morphemes which are not already present in many words [8]. But, does this tendency have an effect on the vocabulary of a language?

In general, reasons behind language changes can be intrinsic ones like the perception, processing and learning of language or extrinsic as in the case of cultural changes [9]. Because of this multitude of factors it is far from trivial to quantitatively unravel the importance of different factors. In the best case, a null model is developed which omits defined factors. Following, it is tested, whether this null model is able to describe observed data or whether a more complex model fits the data significantly better [10]. Here, we perform such a study to analyze vocabulary on the level of morphemes. We focused on ‘accepted’ words, and omit nonce formation [11]. Thereby, we look at two processes simultaneously, the formation of a new word and the acceptance of the new word in the community of speakers.

## Results

### Birth-Death-Innovation Models for morpheme family size distribution

If one follows the life history of a morpheme, it starts with an innovation, i.e. its first emergence in a single word of a language. Following, new words containing the morpheme can be build. At the same time, a word containing the morpheme might be lost from the language. If all words with the morpheme are lost, also the morpheme is lost from the language. In this simple but intuitive model, a morpheme is treated as core unit and no correlation between morphemes is considered. Thus, the model can easily be extended to describe the history of all morphemes of a language.

Analogous processes are widespread in biological systems ranging from population genetics to the evolution of cancer [12]. Intriguingly, already in the beginning of the last century a stochastic framework for their description was developed and named Birth-Death Models or Birth-Death-Innovation Models (BDIMs) [13]–[15]. These models are widely applied in the biological sciences [12]. The BDIMs are discrete Markov processes, i.e. a stochastic process where the state at time t depends on the state at time t-1 alone. If the matrix of state transition probabilities is irreducible and aperiodic, the process has exactly one stationary distribution which is reached by the process within a finite number of steps. Here, we are focusing on the stationary solutions of the processes.

**Figure 1:**
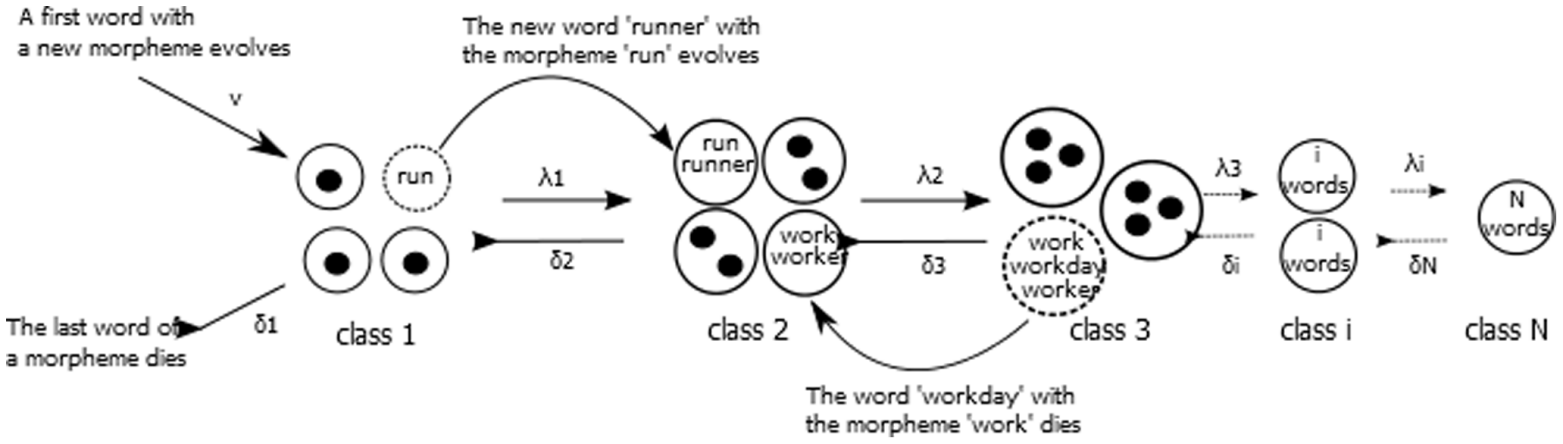
A general scheme of the BDI model for morpheme family distributions. Adopted from [34].

A similar approach was recently used to analyze the distribution of domains (structural, evolutionary and functional parts of proteins) within genomes [16], [17]. This model can be easily adopted to describe the family size distribution of morphemes (meaning bearing parts of words) in a language (Figure 1). Here, the family size is defined as the number of words containing a given morpheme. For example the morpheme ‘work’ might be found in 30 distinct words. Thus, it is the member of the class 30 which contains all morphemes present in 30 words. If a single new word containing the morpheme ‘work’ emerges, for example the word ‘workday’, its class will be changed to 31. Analogously, if one word with the morpheme is lost from the language, the new class would be 29. To each of these processes a rate is assigned – λ_i_, the birth rate, for transition of a morpheme from class i into class i+1 and δ_i_, the death rate, for the transition from class i to class i-1. Finally, the rate of emergence of a new morpheme can be modeled by ν. In the following, we test, whether the family size distribution of morphemes can be modeled by such a BDIM and if yes, how the death and birth rate have to be chosen.

### Fitting BDIMs to morpheme family size distribution

Morpheme family size distributions were calculated for lemmata from different dictionaries and word lists covering about 200 years of English and German. These languages were chosen as they are both Indo-European but differ slightly in their degree of synthesis, i.e. German words tend to contain more morphemes than English ones. As our focus is on word formation, only lemmata (the base form of words) were considered and inflection was deliberately omitted. Each morpheme was assigned to a class according to the number of words it was found in. Finally, the size of each class, i.e. the number of morphemes assigned to the class, was calculated.

**Figure 2:**
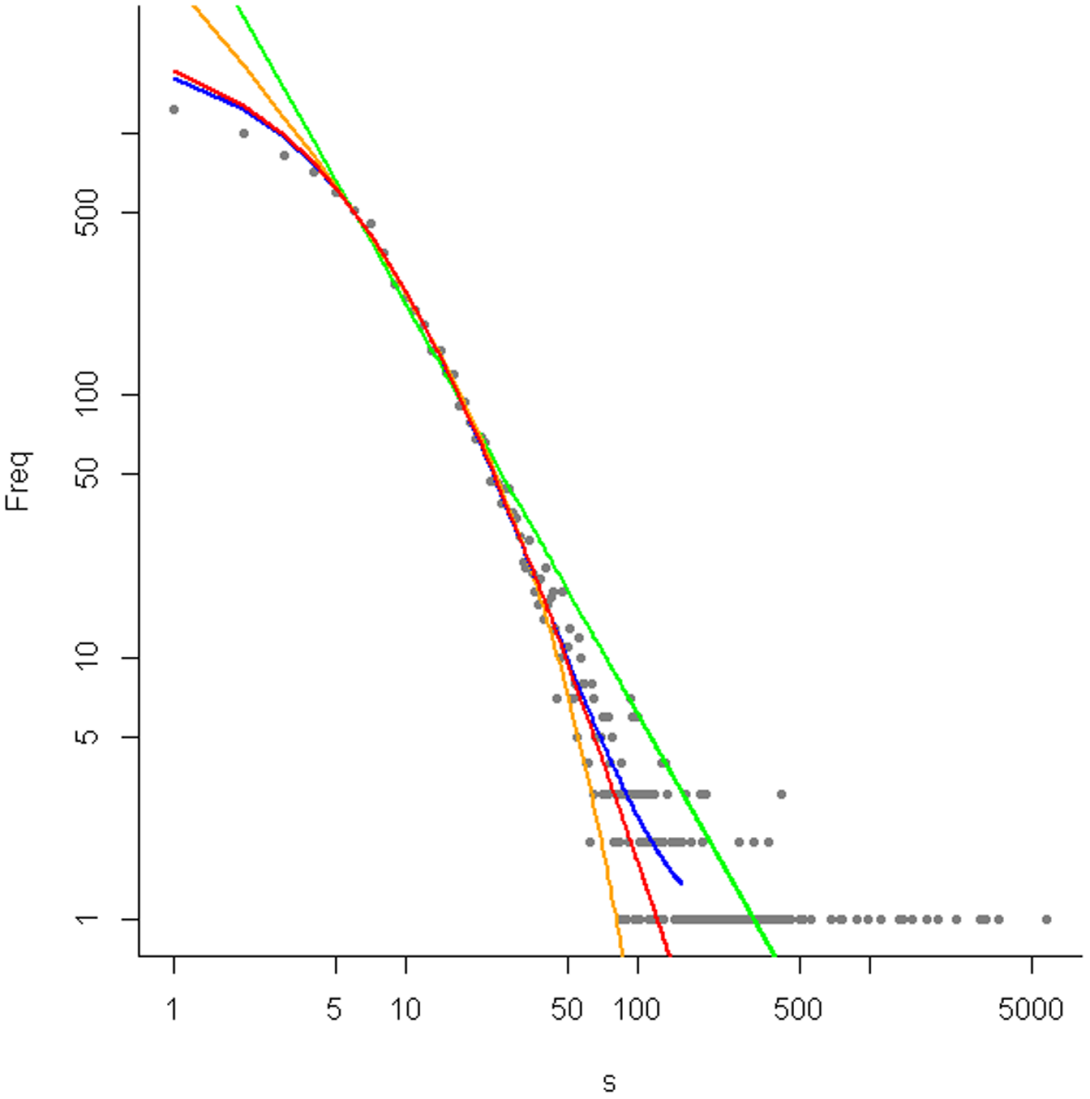
Current English (BNCbaby) with fitted power law (green), simple BDIM (orange), solb BDIM (red) and folb BDIM (blue) to the middle section [5,120]; Word family distribution in double logarithmic scale.

As the simplest model, we fitted the distribution against a general power law, well known in linguistics as Zipfs law or the Yule-Simon distribution. Next, a simple BDIM with birth and death rates independent of the classes was fitted. This model has a proportional relationship between the class number i and the birth/death rate of this class: λ_i_ = λi, δ_i_ = δi (simple BDIM). The innovation rate ν is considered constant. Finally, a generalization of the simple BDIM, the linear BDIM with λ_i_ = λ(i+a) and δ_i_ = δ(i+b) was tested. With positive parameters a and b, both the birth and the death rate per morpheme decrease with increasing class number. We investigated two cases of linear BDIMs: the second order balanced (solb) BDIM does accept λ = δ where the first order balanced (folb) BDIM does not have this restriction. For fitting the models to the data, we omitted the morphemes found in less than six words and in more than an upper limit (Table S1 in File S1). The fitting was performed on normal scale. Figure 2 shows an example of the fitted models; the other word lists are shown in Figures S4 to S8 in File S1. The fit of the models was assessed using the residual sum of squares of the model (RSS) and the result of chi square goodness of fit tests. As the models differ in their number of parameters and are not nested, we furthermore used the Akaike Information Criterion (AIC) and the Bayes Information Criterion (BIC) to measure the fitting of the model to the data. Here, models with more parameters are penalized. Due to the sparsely distributed large word families at the tail, the data needed to be grouped for this analysis into bins with at least 10 morphemes in each bin. This can result in non-monotonic behavior of the model and the data, but is merely an artifact introduced for testing and does not change the data itself, which decay monotonic.

The RSS for all word lists showed the worst fit for the power law and the second worst for the simple BDIM. Both linear models (solb and folb BDIM) showed the same low RSS values and hence the best fit (Figure S1 in File S1). The chi square goodness of fit tests rejected the power law and the simple BDIM for all word lists with highly significant p-values. In contrast, the two linear models were not rejected on a 1% significance level (Table 1). AIC and BIC further supported the choice of the linear BDIMs (Table 1). Thus both the solb and the folb BDIM are suitable models for the family size distribution of morphemes.

**Table 1:** AIC, BIC and P-values of chi square goodness of fit tests for all investigated models.

			Power Law	simple BDIM	solb BDIM	folb BDIM
Adelung	German 18th	AIC	888.02	816.45	**804.40**	805.91
		BIC	896.28	824.71	**815.42**	819.68
		Chi2	<10^−74^	<10^−6^	**0.4865**	0.4201
WDG	German 20th	AIC	1084.84	933.90	**881.98**	882.73
		BIC	1093.58	942.64	**893.63**	897.30
		Chi2	<10^−172^	<10^−12^	**0.1901**	0.0383
BLL	German 20th	AIC	1248.20	1137.54	**1056.08**	1057.12
		BIC	1257.35	1146.69	**1068.28**	1072.37
		Chi2	<10^−75^	<10^−37^	0.2549	**0.3287**
Johnson	English 18th	AIC	727.73	665.38	654.17	**653.36**
		BIC	735.42	673.07	**664.43**	666.18
		Chi2	<10^−48^	<10^−15^	0.0352	**0.0838**
Webster	English beg. 20th	AIC	762.28	650.06	**643.25**	653.36
		BIC	769.97	657.76	**653.51**	666.18
		Chi2	<10^−91^	<10^−14^	0.0156	**0.3621**
BNCbaby	English end 20th	AIC	897.26	779.25	744.72	**744.47**
		BIC	905.52	787.51	**755.73**	758.24
		Chi2	<10^−111^	<10^−6^	**0.9135**	0.7068

For AIC and BIC, lower values mean better fit. In the case of the chi square test, not significant p-values (>0.01) indicate a good fit of the model.Best fitting models are indicated in bold.

To distinguish the solb BDIM from the folb BDIM we analyzed θ = λ/δ. For the solb BDIM, θ was set to 1. Indeed the estimations of θ for all word lists did not differ significantly from 1 as the 95% confidence intervals all covered 1 (Figure S2 in File S1). Furthermore, the estimation of the parameters in the solb BDIM was better than in the folb BDIM, shown by smaller confidence intervals for solb BDIM (Figure S3 in File S1). Together, this indicated that the solb BDIM is more appropriate to describe the morpheme family size distribution.

The estimators for the linear parameters a and b in the solb BDIM range from 3.36 to 7.84 and from 4.18 to 10.35, respectively (Figure S3 in File S1). With positive a and b the average morpheme birth and death rate (normalized to class i) drop with increasing family class from λ+λa and δ+δb for small family class i to λ and δ for large i. For all wordlists, a was smaller than b. This indicates an existing synergy between morphemes in one class [14]. However the confidence intervals are very wide and overlap for a and b. Thus, the difference between the two parameters is not large enough to be proven as statistical significant.

## Discussion

The vocabulary of a language is determined by a multitude of intrinsic and extrinsic factors. Here, we showed that despite these influences a pure stochastic Birth Death Innovation model is sufficient to describe the morpheme family size distribution in German and English as well as in historical data. Obviously, a BDIM is only one of many mechanistic models to generate scale-free distributions. We have recently used a network based representation to analyze the evolution of morphemes in words [8]. A multitude of such networks ranging from the internet to protein interactions have been analyzed. Indeed, their features can be modeled quite well with a preferential attachment approach [18]. Furthermore, many other approaches for the generation of scale-free distributions have been developed. For a review see for example [19].

Here, we decided to adopt BDIMs for modeling as their charm lies in the self-evident interpretability of their parameters. Admittedly, we analyzed only a small set of BDIMs and modifications and refinements of these models are possible. For example, Reed and Hughes used a BDIM to model gene and protein families [20]. Contrasting our model, were new morphemes are drawn from a reservoir of ‘not-yet-invented’ morphemes, here new protein families evolve as a mutation of existing proteins. Indeed this model is well suited for the evolution of protein families. In the case of morphemes, it is arguable whether new morphemes are always derived from existing ones. Still, adding this aspect could enable to model morphemes with more than one meaning, i.e. a new meaning is added to an existing morpheme. In a different application, BDIMs have been used to model surname distributions [21]. This model deviates from the ones analyzed here as the innovation rate is not fixed. Furthermore a sampling effect is considered and the birth rate is modeled as a random variable. These options might be interesting starting points to refine the BDIMs presented here. From a linguistic viewpoint, one could additionally distinguish between derivation and compounding. Currently, the death and birth rates are only depending on the class, not on the type of morpheme. One could argue that derivation is used more frequently and therefore morphemes like ‘-ish’ should be treated differently. In a BDIM one could set different birth and death rates for these two processes. Furthermore, our model assumes a fixed innovation rate, i.e. the rate with which new morphemes are introduced into the vocabulary is constant. More complex models which might correlate the innovation rate to the existing number of morphemes or even words are conceivable. Taken the simplicity of the BDIMs tested here into account, it seems even more surprising that they were sufficient to generate distributions fitting to the data.

More importantly, they provide a mechanistic rather than a phenomenological model for morpheme family size distributions [22]. Therefore the parameters can be directly interpreted. We have shown that the best fitting model was not neutral. In a pure neutral model, the birth and death rates would be independent of the family size. Thus, when building a new word, one would catch a morpheme from a bag containing all morphemes in the same amount as their family size. Deviating from this random model, the data could be better fitted by a second order linear balanced BDIM. Here, absolute terms are added to both, the birth and the death rate. This will have a larger effect for smaller than on larger families. Thus, the birth and death events involve rare morphemes more frequently than in the pure neutral model. This finding is consistent with an analysis of historical language change [8], indicating that our model is indeed capable to describe processes driving language change.

We have already shown that morphemes are well suited to trace cultural changes [8]. Mechanistic models as presented here could enable the statistically sound quantification of these changes. As the birth and death rates are estimated, the probability for a change from class n to class m can be calculated. Thus, rapidly changing morphemes can not only be identified but classified with a p-value in a statistically sound framework [17].

It has to be noted that the morpheme distributions were fitted against the stationary distribution of the BDIM. Although the best fitting models were the same all word lists, the values of the parameters differed (Figure S3 in File S1). This might indicate differences between languages as e.g. the degree of synthesis. Contrasting, it could be a side effect of the used word lists which differ in size and type. It would be interesting to test, whether indeed the parameters a and b change over time and if so whether this change is gradual or in bursts [23]. Our approach would enable to identify and quantify such a historical change in word formation.

Still, the model itself cannot explain why morphemes from smaller families are preferred in word formation. If one assumes an utterance based selection model of language change [24], there are two non-exclusive explanations. First, it could be a bias in word invention. Here, the inventor of a word prefers morphemes which are not yet in too many other words. Second, it could be a bias in word selection. The speaker might try to avoid new words which contain morphemes found in too many other words. So far, we can only speculate about the reason behind this avoidance. Psycholinguistic experiments revealed a positive correlation between morpheme family size and recognition time which would imply an advantage for morphemes from larger families [25], [26]. Still, this effect was attenuated if there are many ‘higher-frequency family members’ [27]. Although our approach does not include frequency, one can assume that large family morphemes have a higher chance of including higher frequency words and are therefore avoided. Furthermore, these studies are based on accepted words and might therefore not capture all aspects related to word formation. One could imagine that if a morpheme is used in many different words with slightly different meanings it will be complicated to identify the correct meaning in the new word. It might be interesting to test whether indeed an individual prefers morphemes from smaller classes in word formation. If that is the case, one might be able to establish a link between an individual's mental representation of a language in the accepted vocabulary of the community speaking this language.

## Materials and Methods

### Word lists

Our analyses cover 200 years of English and German which are related, but slightly different in their degree of synthesis [3], i.e. German has more morphemes per word than English. As we were mainly interested in derivational word-formation, ‘the relationship between lexemes of a word family’ [4], we deliberately omitted inflection (different word forms of a lexem) by using dictionaries and lemmatized word lists. We defined a word as a head entry in a dictionary or as the lemma of the lemmatized corpora. Possible blank characters within a word like in ‘window pane’ were used as morpheme boundaries. The following dictionaries and corpora were used: Johnson – English 18^th^ century [28], Webster – English beginning 20^th^ century [29], BNCbaby – English end 20^th^ century [30], Adelung – German 18^th^ century [28] and WDG – German 20^th^ century [31]. For size of the word and morpheme lists see Table S1 in File S1.

### Morpheme detection

Morphemes were identified automatically by Morfessor version 1.0 [32] with default settings. The decomposition into morphemes was evaluated for 18^th^ century German (Adelung) and 20^th^ century German (WDG), respectively, by comparing the results to a 1% sample of manually decomposed words. 84.37% of the decompositions in WDG were correctly identified with a false positive rate of 15.63% and a false negative rate of 36.15%. In Adelung 85.64% of decompositions were correct with a false positive rate of 14.36% and a false negative rate of 27.44%. In total, 83% of the morphemes in WDG and 86% of those in Adelung were correctly identified. Within the Morpho Challenge 2010, Morfessor 1.0 was evaluated on a gold standard set for English and German with a graph-based assignment algorithm. It reached a precision of 0.8686 and a recall of 0.7226 for English and a precision of 0.8128 and a recall of 0.4806 for German [33].

## Supporting Information

File S1 **This file contains Table S1 and Figures S1–S8.** Figure S1, RSS values for all wordlists and all investigated models. Figure S2, 95%-confidence intervals of *θ = λ/δ* for all wordlists. All confidence intervals cover the value 1. Figure S3, 95%-confidence intervals of the parameters *a* and *b* for solb and folb BDIM. Figure S4, Adelung with fitted power law (green), simple BDIM (orange), solb BDIM (red) and folb BDIM (blue) to the middle section [5,120] Left: Word family distribution in double logarithmic scale Right: Word family distribution grouped into bins for chi square test. Figure S5, WDG with fitted power law (green), simple BDIM (orange), solb BDIM (red) and folb BDIM (blue) to the middle section [5,140] Left: Word family distribution in double logarithmic scale Right: Word family distribution grouped into bins for chi square test. Figure S6, BLL with fitted power law (green), simple BDIM (orange), solb BDIM (red) and folb BDIM (blue) to the middle section [5,160] Left: Word family distribution in double logarithmic scale Right: Word family distribution grouped into bins for chi square test. Figure S7, Johnson with fitted power law (green), simple BDIM (orange), solb BDIM (red) and folb BDIM (blue) to the middle section [5,100] Left: Word family distribution in double logarithmic scale Right: Word family distribution grouped into bins for chi square test. Figure S8, Webster with fitted power law (green), simple BDIM (orange), solb BDIM (red) and folb BDIM (blue) to the middle section [5,100] Left: Word family distribution in double logarithmic scale Right: Word family distribution grouped into bins for chi square test. Table S1, Number of words and morphemes in the word lists and upper border of family sizes used for the fitting to the models.
(DOCX)
