# Supplementary material for: Word Formation Is Aware of Morpheme Family Size
Source: PLoS One. 2014 Apr 4;9(4):e93978. doi: 10.1371/journal.pone.0093978 (PMC3976386; doi:10.1371/journal.pone.0093978)
Supplement: File S1 — This file contains Table S1 and Figures S1–S8. Figure S1, RSS values for all wordlists and all investigated models. Figure S2, 95%-confidence intervals of θ = λ/δ for all wordlists. All confidence intervals cover the value 1. Figure S3, 95%-confidence intervals of the parameters a and b for solb and folb BDIM. Figure S4, Adelung with fitted power law (green), simple BDIM (orange), solb BDIM (red) and folb BDIM (blue) to the middle section [5,120] Left: Word family distribution in double logarithmic scale Right: Word family distribution grouped into bins for chi square test. Figure S5, WDG with fitted power law (green), simple BDIM (orange), solb BDIM (red) and folb BDIM (blue) to the middle section [5,140] Left: Word family distribution in double logarithmic scale Right: Word family distribution grouped into bins for chi square test. Figure S6, BLL with fitted power law (green), simple BDIM (orange), solb BDIM (red) and folb BDIM (blue) to the middle section [5,160] Left: Word family distribution in double logarithmic scale Right: Word family distribution grouped into bins for chi square test. Figure S7, Johnson with fitted power law (green), simple BDIM (orange), solb BDIM (red) and folb BDIM (blue) to the middle section [5,100] Left: Word family distribution in double logarithmic scale Right: Word family distribution grouped into bins for chi square test. Figure S8, Webster with fitted power law (green), simple BDIM (orange), solb BDIM (red) and folb BDIM (blue) to the middle section [5,100] Left: Word family distribution in double logarithmic scale Right: Word family distribution grouped into bins for chi square test. Table S1, Number of words and morphemes in the word lists and upper border of family sizes used for the fitting to the models. (DOCX) [file pone.0093978.s001.docx]

## Supporting Information


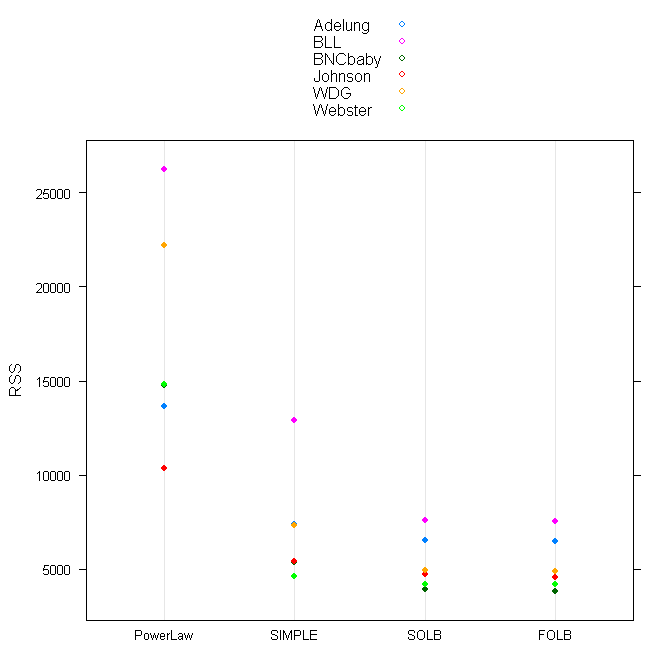


**Figure S1:** RSS values for all wordlists and all investigated models


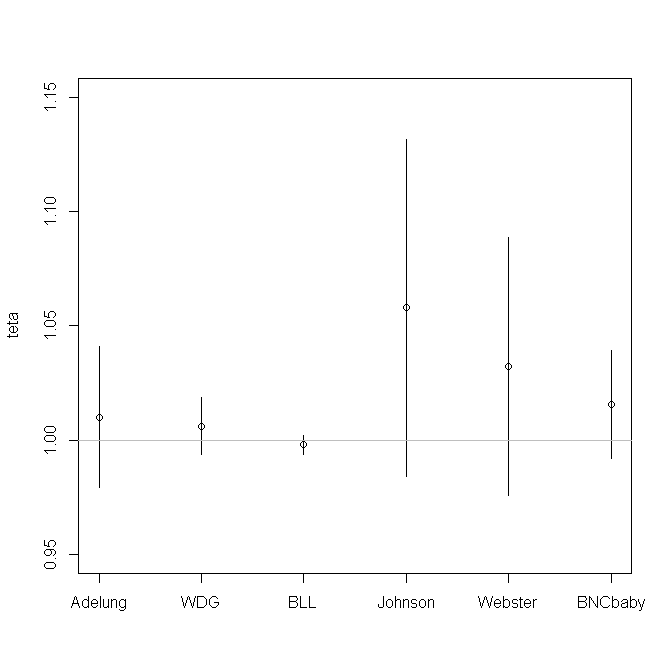


**Figure S2:** 95%-confidence intervals of *θ=λ/δ* for all wordlists. All confidence intervals cover the value 1.


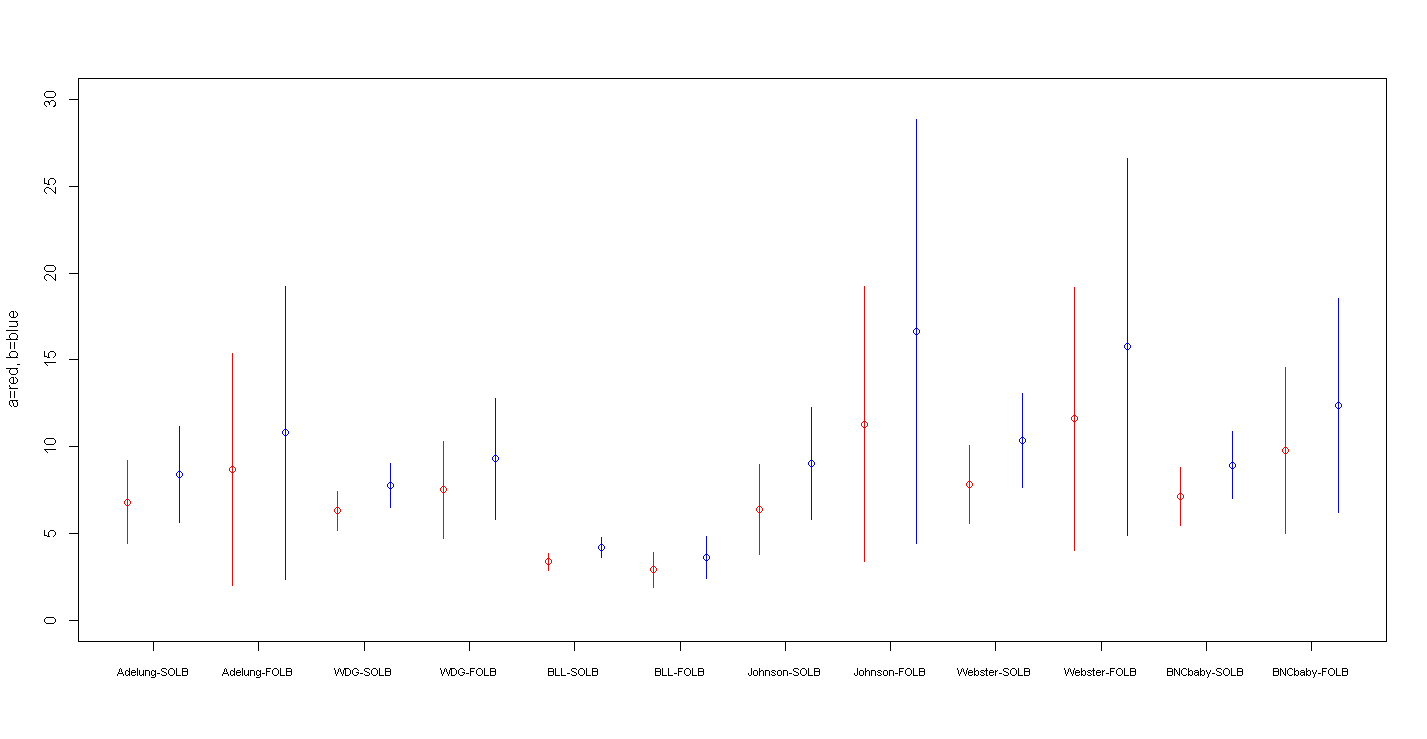


**Figure S3:** 95%-confidence intervals of the parameters *a* and *b* for solb and folb BDIM.

**
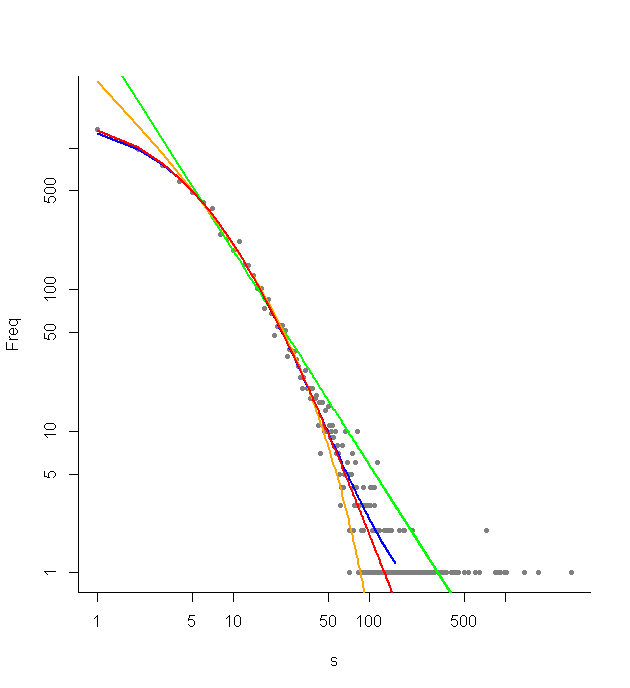
**

**Figure S4:** Adelung with fitted power law (green), simple BDIM (orange), solb BDIM (red) and folb BDIM (blue) to the middle section [5,120] **Left:** Word family distribution in double logarithmic scale **Right:** Word family distribution grouped into bins for chi square test

**
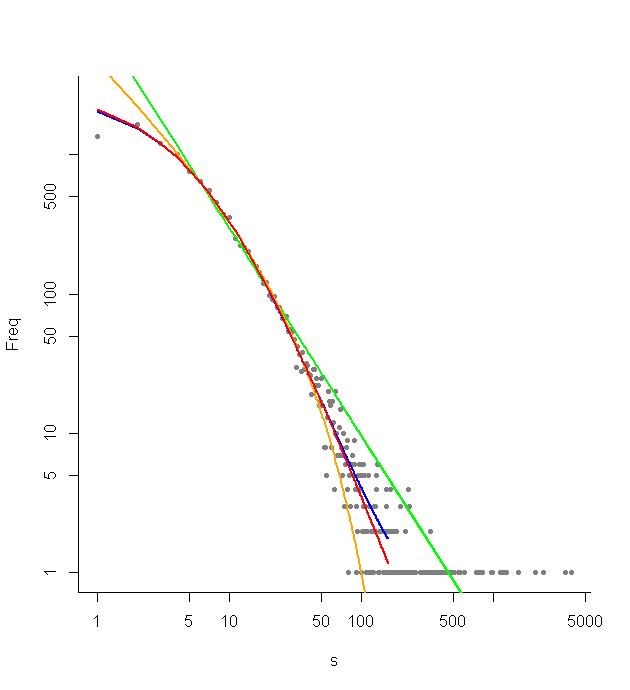
**

**Figure S5:** WDG with fitted power law (green), simple BDIM (orange), solb BDIM (red) and folb BDIM (blue) to the middle section [5,140] **Left:** Word family distribution in double logarithmic scale **Right:** Word family distribution grouped into bins for chi square test

**
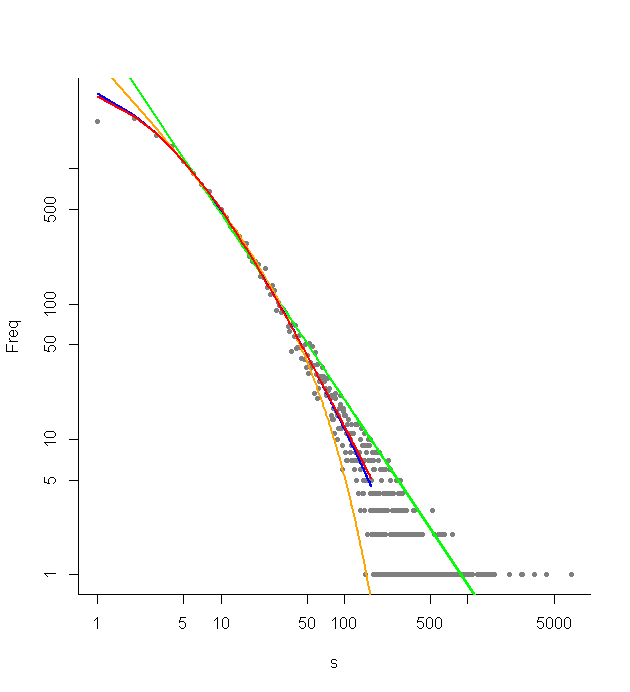
**

**Figure S6:** BLL with fitted power law (green), simple BDIM (orange), solb BDIM (red) and folb BDIM (blue) to the middle section [5,160] **Left:** Word family distribution in double logarithmic scale **Right:** Word family distribution grouped into bins for chi square test

**
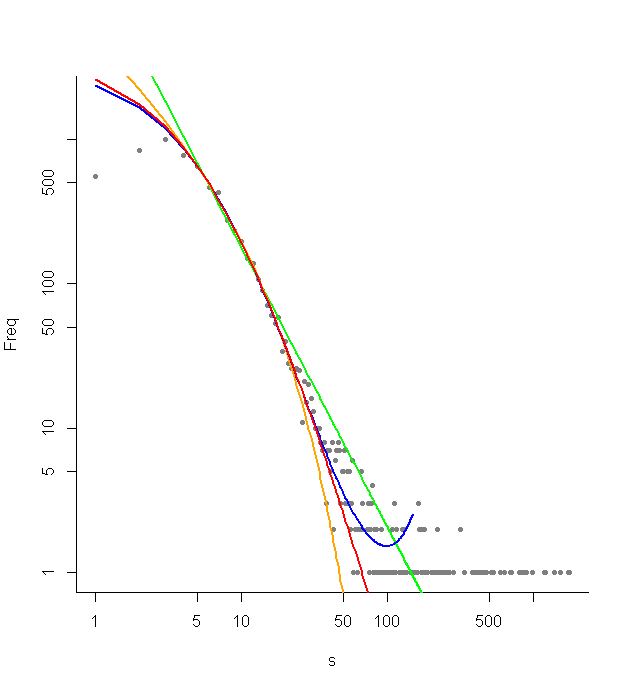
**

**Figure S7:** Johnson with fitted power law (green), simple BDIM (orange), solb BDIM (red) and folb BDIM (blue) to the middle section [5,100] **Left:** Word family distribution in double logarithmic scale **Right:** Word family distribution grouped into bins for chi square test

**
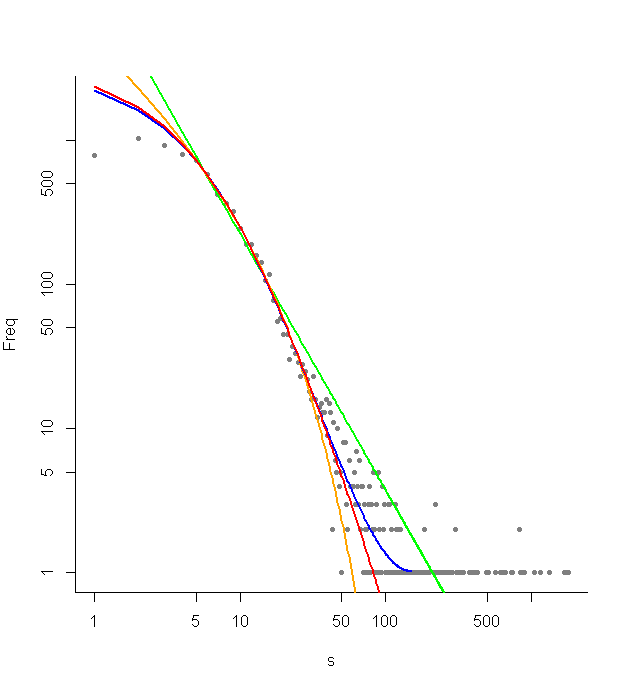
**

**Figure S8:** Webster with fitted power law (green), simple BDIM (orange), solb BDIM (red) and folb BDIM (blue) to the middle section [5,100] **Left:** Word family distribution in double logarithmic scale **Right:** Word family distribution grouped into bins for chi square test

**Table S1:** Number of words and morphemes in the word lists and upper border of family sizes used for the fitting to the models.

|  | Number of words | Number of morphemes | Upper border |
| --- | --- | --- | --- |
| Adelung | 54,663 | 7,865 | 120 |
| WDG | 86,129 | 11,996 | 140 |
| BLL | 254,159 | 19,935 | 160 |
| Johnson | 37,588 | 6,725 | 100 |
| Webster | 45,236 | 8,104 | 100 |
| BNCbaby | 63,077 | 8,646 | 120 |
